# Evaluation of treatment compliance in gout patients: a patient-centered study

**DOI:** 10.55730/1300-0144.5985

**Published:** 2025-01-08

**Authors:** M. Buğra GÖRGÜLÜ, Rıza Can KARDAŞ, Dilara KOÇ ŞERAMET, Rıdvan MERCAN, Mehmet Engin TEZCAN, Abdurrahman TUFAN, Hamit KÜÇÜK, Berna GÖKER, M. Akif ÖZTÜRK

**Affiliations:** 1 Department of Internal Medicine, Faculty of Medicine, Gazi University, Ankara, Turkiye; 2 Division of Rheumatology, Department of Internal Medicine, Faculty of Medicine, Gazi University, Ankara, Turkiye; 3 Department of Internal Medicine, Faculty of Medicine, Namık Kemal University, Tekirdağ, Turkiye; 4 Division of Rheumatology, Department of Internal Medicine, Faculty of Medicine, Namık Kemal University, Tekirdağ, Turkiye; 5 Rheumatology Clinic, City Hospital, Kartal Lütfi Kırdar City Hospital, İstanbul, Turkiye

**Keywords:** Gout, treatment compliance, patient education

## Abstract

**Background/aim:**

Most studies on unsuccessful gout treatment suggest that knowledge gaps and inadequate physician interventions are major contributors. However, there is a lack of research on the extent to which patients, educated by knowledgeable and experienced physicians, adhere to these recommendations.

**Materials and methods:**

This study evaluated patients seen in university rheumatology clinics who were adequately informed about diet, target serum uric acid levels, and gout by rheumatologists. We assessed their compliance with treatment, clinical and laboratory findings, and disease status a median of seven years after treatment initiation. A total of 302 gout patients who began treatment in tertiary rheumatology centers and received adequate information were screened. After the initial interview, 195 patients met the study criteria and were included. Treatment compliance was evaluated based on self-reports, and target uric acid level achievement was assessed using medical records.

**Results:**

Of the 195 patients included in the study, 87.4% were male, with a median age of 59 years. Common comorbidities included hypertension (50%), hypertriglyceridemia (54.5%), and diabetes mellitus (23.2%). The median BMI was 29.3 kg/m^2^, with 45.1% classified as overweight and 44.6% as obese. At the last follow-up, 68.5% of patients who continued their prescribed medication remained adherent. Nonadherent patients consumed significantly more meat (≥3.5 servings/week) and experienced longer intervals between flares. Nonadherent patients also had higher uric acid levels (7.25 mg/dL vs. 6.0 mg/dL, p < 0.001) and more frequent gout flares. Regular follow-up visits were significantly lower in nonadherent patients.

**Conclusion:**

Achieving an acceptable level of treatment adherence and success in gout patients depends on adequate disease knowledge and appropriate education provided by physicians.

## 1. Introduction

Gout is a potentially treatable disease. With effective treatment, acute exacerbations of arthritis can be prevented, and even tophi may disappear. Current guidelines for its treatment emphasize the importance of patient compliance with a recommended diet, lifestyle changes, and medical treatment. However, studies have shown that only a small proportion of patients with gout receive adequate medical advice and treatment. Inadequate follow-up and noncompliance with recommended treatment make gout difficult to manage for both patients and doctors. Physicians often focus on managing acute attacks rather than treating gout as a chronic disease characterized by progressive crystal deposition [1]. Uric acid-lowering therapy is frequently not prescribed for a sufficient duration, and patients often receive inadequate doses [1].

Gout is primarily managed by general practitioners in primary care, and most patients are never referred for specialist consultation [2]. The approach to gout treatment by general practitioners is often inaccurate or inadequate, with studies showing that some common misconceptions are still practiced [3–6]. For instance, a study conducted in Türkiye demonstrated that gout treatment in primary health care centers was suboptimal and long-term management was inadequate [7]. Patient compliance with treatment is also a significant issue. More than half of the patients do not comply with uric acid-lowering treatment and discontinue treatment within 12 months [1]. An educational study combining treatment compliance with regular monitoring of uric acid levels found that the target uric acid level was reached in 90 percent of patients [1,8].

A multidisciplinary approach involving diet, lifestyle modifications, and pharmacological treatment is effective in managing gout. While existing research has primarily addressed physicians knowledge gaps and clinical practices, patient perceptions and adherence to recommended therapies remain understudied. This study aimed to address this gap by evaluating treatment adherence among gout patients who received comprehensive disease education at tertiary rheumatology centers. Patients adherence to clinical recommendations, achievement of target serum urate levels, and the relationship between adherence and clinical outcomes were assessed.

## 2. Materials and methods

### 2.1. Patient selection

This multicenter study included patients with previously known or newly diagnosed gout admitted to Gazi University Hospital, Kartal Dr. Lütfi Kırdar City Hospital, and Tekirdağ Namık Kemal University Hospital in Türkiye. Patients with gout were included according to the 2015 American College of Rheumatology/European League Against Rheumatology (ACR/EULAR) gout classification criteria [9]. All of the centers are tertiary rheumatology clinics, where detailed education about the disease and its treatment is provided to patients with gout as a standard approach.

Patients were included if they met the following criteria: (1) provided written informed consent, (2) were over 18 years of age, and (3) satisfied the ACR/EULAR gout classification criteria. Patients were excluded if they had: (1) an inability to communicate, (2) currently used antidepressants, (3) a gout diagnosis within the last year, or (4) unavailable laboratory results in medical records or the national database (“E-nabız”).

### 2.2. Data collection

Data were collected through a retrospective review of electronic medical records and a self-administered questionnaire. The questionnaire queried patients about their adherence to gout treatment medications (urate-lowering therapy (ULT) and colchicine). Medical records were reviewed to extract data on demographics (age, sex, and body mass index), gout diagnosis date, comorbidities, medications prescribed, laboratory results (serum uric acid levels), and documented gout attacks during the follow-up period. Adherence was defined as using a gout treatment medication at the time of administration of the questionnaire. Target serum urate level achievement was defined as <6.0 mg/dL without risk factors and <5.0 mg/dL with risk factors, according to EULAR recommendations [10].

### 2.3. Statistical analysis

All data analyses were performed using SPSS version 22. Numerical parameters were expressed as mean and standard deviation if they were parametrically distributed and median, minimum, and maximum values if they were nonparametrically distributed. The difference between independent groups with parametric and nonparametric distribution was evaluated using Student’s t-test. The difference between two dependent groups was evaluated using the Wilcoxon test if at least one was nonparametrically distributed, or the paired t-test if both groups were parametric. Pearson and Spearman correlation tests were used to show the relationship between variables depending on the type and distribution of the data. The chi-square test was used for categorical parameters. Probability values of p < 0.05 were considered significant. Finally, univariate and multivariate logistic regression models were used to determine odds ratios. The backward likelihood ratio method was used in multivariate logistic regression.

## 3. Results

### 3.1. Patient characteristics

We identified 302 patients diagnosed and treated for gout in three tertiary rheumatology clinics. After applying the inclusion and exclusion criteria, data from 195 patients were analyzed. All patients fulfilled the 2015 ACR/EULAR gout classification criteria. Medical record review determined a median follow-up and treatment duration for gout of seven years (IQR: 1–15 years).

Table 1 summarizes the baseline characteristics of the study population. Most patients were male (87.4%, n = 173), with a median age of 59 (range: 24–86 years). All female patients (n = 22) were postmenopausal and had a median age at diagnosis of 66.5 (47–84 years), compared to 58 in men (range: 24–86 years).

Common comorbidities included hypertension (50%, n = 99), hypertriglyceridemia (54.5%, n = 108), and diabetes mellitus (23.2%, n = 46). The median body mass index (BMI) was 29.3 kg/m^2^ (range: 21.6–47.18 kg/m^2^), with a significant proportion of patients classified as overweight (45.1%) or obese (44.6%). Women had a significantly higher median BMI than men (32.8 kg/m^2^ vs. 29.05 kg/m^2^, p = 0.003).

### 3.2. Treatment adherence and outcomes

A median follow-up of seven years (IQR: 1–15 years) revealed that the most common treatment was allopurinol with colchicine (46.7%), followed by allopurinol alone (25.1%, Table 2). Colchicine monotherapy was used by 10.3%, while febuxostat, either by itself or with colchicine, was used by 8.2% of the patients (4.1% each). Additionally, 9.7% of the patients were not on any medication.

Among patients taking medication at their last follow-up, 68.5% (122 patients) who continued taking their prescribed medication remained adherent at their last follow-up ( Table 3). There were no significant differences in baseline characteristics and comorbidities between adherent and nonadherent patients. However, nonadherent patients consumed significantly more meat (≥3.5 servings/week) compared to adherent patients (51.9% vs. 26.2%, p = 0.002). Additionally, they experienced longer intervals between flares (median 5 months vs. 3 months, p = 0.028). Laboratory parameters (uric acid, CRP, and creatinine) at the first flare were similar between the groups.

**Figure 1:**
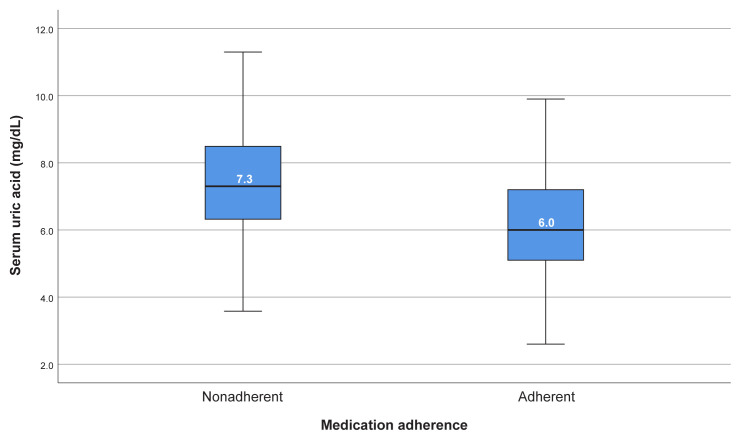
Uric acid levels at the last follow-up.

**Figure 2:**
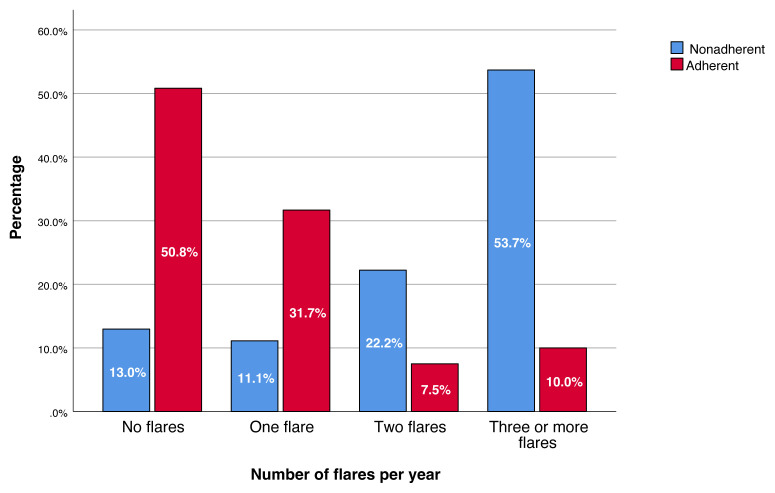
Distribution of flare frequencies.

Analysis of follow-up data revealed significantly lower allopurinol use (18.5% vs. 32.0%, p < 0.001) and higher colchicine monotherapy (25.9% vs. 4.9%, p < 0.001) in the nonadherent patients ( Table 4). Regular follow-up visits were markedly lower in this group (1.9% vs. 25.6%, p < 0.001), with no difference in rates for reasons of nonattendance. Nonadherent patients also had higher median uric acid levels (7.25 mg/dL vs. 6.0 mg/dL, p < 0.001) ( Figure 1) and a lower proportion achieving target levels (<6 mg/dL, 18.5% vs. 49.2%, p < 0.001). They experienced significantly more gout flares within the past year (median 3 vs. 0, p < 0.001), with a greater proportion having three or more flares (53.7% vs. 10.0%, p < 0.001) ( Figure 2). Interestingly, gout-inducing medication use was more common in adherent patients (41.7% vs. 21.3%, p = 0.017). No significant differences were observed in other medications (diuretics, acetylsalicylic acid, and ACE inhibitor/ARB), inflammatory markers (CRP), kidney function (creatinine), or reported triggers (diet nonadherence and alcohol use).

### 3.3. Reasons for nonadherence and nonattendance

Excluding drug intolerance or adverse events, nonadherent patients (n = 54) most frequently reported using medication only during flares (50%) or due to forgetfulness/lack of perceived need (44.4%) ( Table 5). Ineffectiveness was a less common reason (5.6%).

Patients achieving treatment goals (serum uric acid [SUA] < 6 mg/dL, n = 77) exhibited significantly different nonattendance patterns compared to those not achieving goals (SUA ≥ 6 mg/dL, n = 117, Table 6 ). Those achieving treatment goals reported higher rates of regular attendance/nonattendance (40.3% vs. 10.3%, p < 0.05). Conversely, perceived disease improvement was expressed by 20.5% of patients not at goal, compared to none in the treatment goal group (p < 0.05). No significant differences were observed for low perceived disease activity or COVID-19 restrictions.

## 4. Discussion

Gout is a treatable condition, but optimal management remains challenging due to suboptimal adherence to recommended treatment strategies [11,12]. Key factors contributing to this issue include inadequate patient education, infrequent follow-up visits, and inconsistent medication use [10,13,14]. While general practitioners oversee most gout cases, their management may be constrained by limited gout-specific training. Even within rheumatology clinics—settings typically recognized for expertise in chronic disease management—variation in the consistent recommendation and implementation of ULT has been observed [1,5,7,8].

Of the 302 gout patients contacted, 198 met the inclusion criteria and were enrolled in the study. The cohort consisted of 12.6% females (n = 25) and 87.4% males (n = 173), with a median age of 59 years (24–86 years). All postmenopausal females had a significantly higher median age (66.5 years) than the males (p < 0.001). These demographic characteristics are consistent with the existing literature on gout epidemiology [15–19], suggesting that the study population is representative.

Following treatment, serum uric acid levels significantly decreased (p < 0.001), with the median level dropping from 8.85 mg/dL (range: 6.0–15.0 mg/dL) to 6.5 mg/dL (2.1–13.2 mg/dL). This reduction aligns with expectations, as some participants adhered to recommended gout management strategies before enrollment. These findings reinforce the critical role of ULT in achieving effective disease control, as established in prior research [10–13].

Despite the observed benefits of ULT, only 68.5% of patients (122/178) at follow-up reported adherence to their prescribed regimen. Allopurinol was the most commonly used ULT, often in combination with colchicine. However, 10.6% of patients relied solely on colchicine, a practice not typically recommended for continuous prophylaxis, potentially reflecting nonadherence to physician recommendations. This suboptimal adherence underscores the significant challenges in maintaining effective gout management and aligns with prior findings linking nonadherence to poor long-term outcomes [27].

Our study highlights the persistent challenges associated with medication adherence and patient engagement in gout management. Strategies aimed at educating patients about urate control and providing clear, actionable medical advice have proven effective in improving outcomes [28,29]. For instance, a study by Michael et al. demonstrated that allowing patients to self-monitor serum urate levels supports adherence to allopurinol, enhances urate control, and reduces gout flares [30]. Additionally, addressing pessimistic disease perceptions has been shown to encourage prudent self-management behaviors and improve clinical outcomes [31].

High adherence to ULT was sustained among patients attending regular rheumatology follow-ups (median follow-up: 7 years). Regular follow-up visits were significantly associated with achieving target serum urate levels (<6.8 mg/dL). Nonadherent patients were 4.1 times more likely to fail to reach target urate levels (p = 0.001). While these findings emphasize the importance of follow-up care, it is important to acknowledge that self-reported adherence data may be subject to recall bias. Future studies should incorporate objective measures to corroborate patient-reported adherence.

Both medication adherence and regular follow-up attendance were strong predictors of treatment success. Among adherent patients, 49.2% achieved target uric acid levels, compared to only 18.5% of nonadherent patients (p < 0.001). Similarly, patients who consistently attended follow-up visits had a significantly higher rate of achieving target uric acid levels (90.6%) compared to those who missed appointments (29.4%) (p < 0.001). Notably, missing follow-up visits increased the risk of failing to achieve target levels by more than 21-fold. These findings underscore the critical role of consistent follow-up care in achieving optimal outcomes.

Treatment adherence significantly influenced clinical and laboratory outcomes. Adherent patients had lower mean serum uric acid levels at their last follow-up (6.19 ± 1.69 mg/dL) compared to nonadherent patients (7.43 ± 1.83 mg/dL; p < 0.001). Furthermore, the median annual frequency of gout attacks was significantly lower in adherent patients (0; range: 0–10) than in nonadherent patients (3; range: 0–25; p < 0.001). Despite these positive outcomes, nearly half (50.8%) of adherent patients did not reach the target serum uric acid level, which highlighted the need for individualized dose adjustments and ongoing monitoring by physicians to optimize treatment outcomes.

Among the nonadherent patients (n = 54), the most frequently cited reasons for nonadherence were forgetting to take medications, disliking them (35.7%), and underestimating the severity of gout’s impact on daily life (48.6%). These patients were significantly less likely to use allopurinol, the recommended long-term ULT, and more likely to rely on colchicine monotherapy, which is not intended for continuous prophylaxis. Additionally, nonadherent patients attended fewer follow-up visits and experienced more frequent gout flares, further exacerbating disease outcomes.

Treatment goals (serum uric acid <6 mg/dL) were strongly associated with clinic attendance patterns. Patients who achieved these goals reported higher engagement, including regular attendance and consistent follow-up reporting, than those who did not. Interestingly, perceived improvement often led some patients to reduce their attendance frequency, potentially undermining sustained disease control. This finding emphasizes the importance of continuous education on the need for adherence and monitoring, regardless of perceived improvement in symptoms.

Consistent with prior research linking obesity to gout risk [20,21], most participants (89.7%) had a BMI exceeding normal limits, with a median BMI of 29.3 (range: 21.6–47.18). Women exhibited a significantly higher median BMI than men (32.8 vs. 29.05; p = 0.003). The prevalence of obesity (44.6%) in our cohort aligns with findings from a previous Turkish study (40.1%) [18]. Additionally, our study revealed a high prevalence of comorbidities, including hypertension (50.0%), hypertriglyceridemia (54.5%), and diabetes mellitus (23.2%). These findings reflect established associations between gout and comorbid conditions [22–24]. Differences in comorbidity prevalence across studies likely stem from variations in ethnicity, lifestyle factors, and baseline health characteristics [25,26].

The first metatarsophalangeal joint was the most common site of initial gout attacks in our cohort (71.9%), followed by the ankle (19.9%), with knee involvement being relatively rare (1.5%). These findings are consistent with previously reported patterns of gout attack localization [18,26]. The interval between the first two gout attacks ranged from 1 to 24 months (median: 3 months), reflecting the frequent recurrence of early gout episodes observed in prior studies.

Some patients experienced gout attacks despite serum uric acid levels below 6 mg/dL, a phenomenon inconsistent with typical expectations [22–24]. These patients reported poor adherence to medication regimens and often initiated ULT only during acute attacks. This aligns with prior research suggesting rapid uric acid reduction without colchicine prophylaxis can trigger flares [1,2,8]. Intermittent medication use, coupled with preexisting health conditions, likely contributed to these unexpected gout attacks.

Our study has several limitations. The lack of detailed data on certain clinical features, such as the presence of tophi or the specific etiologies of gout flares, restricted a more comprehensive analysis. Additionally, conducting interviews via telephone precluded the use of standardized tools for assessing patient-reported outcomes, such as the SF-36 and HAQ-DI. Future studies should address these gaps by incorporating detailed clinical evaluations and validated assessment tools.

In conclusion, our study underscores the critical role of treatment adherence and regular follow-up visits in achieving optimal gout management. Patients who adhered to physician-recommended medication regimens and maintained consistent follow-up attendance demonstrated significantly lower serum uric acid levels and fewer gout attacks. These findings align with existing literature emphasizing the importance of ULT and comprehensive patient management for effective disease control [22,24]. Strategies to enhance treatment compliance, including patient education and behavioral interventions, are essential for improving long-term outcomes in gout management.

## Figures and Tables

**Table 1:** Baseline characteristics of the study population.

	Gout patients (n = 195)
**Sex**, n (%)	
Male	173 (87.4)
Female	22 (12.6)
**Age at diagnosis**, years, median (IQR)	
Male	58.0 (19)
Female	66.5 (12)
**Comorbidities**, n (%)	
Hypertension	99 (50.0)
Hypertriglyceridemia	108 (54.5)
Diabetes mellitus	46 (23.2)
Coronary artery disease	43 (21.7)
Concomitant rheumatic disease	20 (10.1)
Renal calculi	45 (22.7)
Chronic kidney disease	28 (14.5)
**BMI**, kg/m^2^, median (IQR)	29.3 (5.7)
**BMI category**, n (%)	
Normal (20–24.99 kg/m^2^)	20 (10.3)
Overweight (25–29.99 kg/m^2^)	88 (45.1)
Obese (≥30 kg/m^2^)	87 (44.6)
Class I (30–34.99 kg/m^2^)	57 (29.2)
Class II (35–39.99 kg/m^2^)	21 (10.8)
Class III (≥40 kg/m^2^)	9 (4.6)

**Table 2:** Gout treatment at last follow-up.

Medication, n (%)	Gout patients (n = 195)
**Overall**	
ULT	156 (80)
Allopurinol	140 (71.8)
Febuxostat	16 (8.2)
Colchicine	119 (61)
None	19 (9.7)
**Combined use**	
Allopurinol and colchicine	91 (46.7)
Allopurinol alone	49 (25.1)
Febuxostat and colchicine	8 (4.1)
Febuxostat alone	8 (4.1)
Colchicine alone	20 (10.3)

**Table 3:** Demographic, social, and clinical characteristics of adherent vs. nonadherent patients.

	Adherent (n = 122)	Nonadherent (n = 54)	p
**Demographics and comorbidities**			
Male sex, n (%)	105 (86.1)	48 (88.9)	0.809
Age, years, mean ± SD	59.3 ± 12.7	55.9 ± 13.7	0.137
Obesity, n (%)	46 (37.7)	23 (42.6)	0.616
Diabetes, n (%)	28 (23.0)	11 (20.4)	0.844
Hypertension, n (%)	66 (54.1)	24 (44.4)	0.256
Coronary artery disease, n (%)	28 (23.0)	12 (22.2)	1.000
Urolithiasis, n (%)	23 (18.9)	15 (27.8)	0.233
Chronic kidney disease, n (%)	23 (19.0)	4 (7.5)	0.07
**Social and lifestyle factors**, n (%)			
Alcohol use	7 (5.7)	5 (9.3)	0.517
Smoking	40 (32.8)	19 (35.2)	0.863
Meat consumption ≥3.5 portion/week	32 (26.2)	28 (51.9)	**0.002**
**Characteristics of the first flare**			
Age at first flare, years, mean ± SD	50.0 ± 13.9	47.5 ± 14.2	0.291
Duration between first two flares, months, median (IQR)	3.0 (5)	5.0 (4)	**0.028**
**Laboratory at the first flare**			
Uric acid, mg/dL, median (IQR)	9.1 (1.66)	8.6 (1.67)	0.138
CRP, mg/L, median (IQR)	7.14 (12.4)	8.7 (11.8)	0.325
Creatinine, mg/dL, median (IQR)	0.99 (0.33)	0.99 (0.31)	0.565
eGFR (2021 CKD-EPI), mL/min/1.73 m^2^, median (IQR)	91 (35)	93 (34)	0.197

**Table 4:** Clinical management and outcomes at the last follow-up.

	Adherent (n = 122)	Nonadherent (n = 54)	p
**Non-gout treatment at follow-up**, n (%)			
Diuretics	27 (22.1)	7 (13)	0.214
Acetylsalicylic acid	26 (21.5)	8 (14.8)	0.408
ACE inhibitor/ARB	53 (43.4)	16 (29.6)	0.096
**Gout treatment at follow-up**, n (%)			
Allopurinol	39 (32)	10 (18.5)	**<0.001**
Febuxostat	7 (5.7)	1 (1.9)	
Colchicine monotherapy	6 (4.9)	14 (25.9)	
Colchicine and allopurinol	62 (50.8)	29 (53.7)	
Colchicine and febuxostat	8 (6.6)	0	
**Attendance pattern**, n (%)			
Regular follow-up	31 (25.6)	1 (1.9)	**<0.001**
Irregular follow-up	90 (74.4)	53 (98.1)	
**Nonattendance**, n (%)			
Not reported/regular attendance	35 (28.7)	8 (14.8)	0.09
Low perceived disease activity	35 (28.7)	14 (25.9)	
COVID-19 pandemic restrictions	35 (28.7)	25 (46.3)	
Perceived disease improvement	17 (13.9)	7 (13.0)	
**Laboratory at last follow-up**			
CRP, mg/L, median (IQR)	4.0 (5.1)	3.3 (6.5)	0.973
Creatinine, mg/dL, median (IQR)	0.99 (0.32)	1.00 (0.29)	0.800
eGFR (2021 CKD-EPI), mL/min/1.73 m^2^, median (IQR)	83 (36)	91 (28)	0.161
Uric acid, median (IQR)	6.0 (2.2)	7.25 (2.3)	**<0.001**
Serum uric acid <6 mg/dL, n (%)	60 (49.2)	10 (18.5)	**<0.001**
**Flares in previous years**			
Number of gout flares, median (IQR)	0 (1)	3 (2)	**<0.001**
No flares, n (%)	61 (50.8)	7 (13.0)	**<0.001**
Single flare, n (%)	38 (31.7)	6 (11.1)	
Two flares, n (%)	9 (7.5)	12 (22.2)	
≥3 flares, n (%)	12 (10.0)	29 (53.7)	
**Triggers for flares**, n (%)			
Diet nonadherence	91 (88.3)	40 (87.0)	0.791
Alcohol	11 (10.7)	7 (14.9)	0.588
Gout-inducing medication use	43 (41.7)	10 (21.3)	**0.017**

ACE inhibitor: angiotensin-converting enzyme inhibitor, ARB: angiotensin-II receptor blocker

**Table 5:** Reasons for medication nonadherence excluding drug intolerance or adverse events by SUA level.

	SUA <6 mg/dL (n = 10)	SUA ≥6 mg/dL (n = 44)	p
**Reasons for nonadherence**, n (%)			
Using medication only during flares	4 (40)	23 (52.3)	0.681
Forgetfulness/lack of perceived need	5 (50)	19 (43.2)	
Ineffectiveness	1 (1)	2 (4.5)	

**Table 6:** Reasons for nonattendance by SUA level.

	SUA <6 mg/dL (n = 77)	SUA ≥6 mg/dL (n = 117)	p
**Nonattendance**, n (%)			
Not reported/regular attendance	31 (40.3)	12 (10.3)	**<0.001**
Low perceived disease activity	26 (33.8)	39 (33.3)	
COVID-19 pandemic restrictions	20 (26.0)	42 (35.9)	
Perceived disease improvement	0	24 (20.5)	
